# Antinociceptive Activities of the Methanolic Extract of the Stem Bark of* Boswellia dalzielii* Hutch. (Burseraceae) in Rats Are NO/cGMP/ATP-Sensitive-K^+^ Channel Activation Dependent

**DOI:** 10.1155/2017/6374907

**Published:** 2017-12-06

**Authors:** Marius Mbiantcha, Alain Ngouonpe Wembe, Amadou Dawe, William Yousseu Nana, Gilbert Ateufack

**Affiliations:** ^1^Department of Animal Biology, Faculty of Science, University of Dschang, P.O. Box 67, Dschang, Cameroon; ^2^Department of Chemistry, Higher Teachers' Training College, University of Yaoundé l, P.O. Box 47, Yaoundé, Cameroon; ^3^Department of Chemistry, Higher Teachers' Training College, University of Maroua, P.O. Box 55, Maroua, Cameroon

## Abstract

*Boswellia dalzielii (B. dalzielii)* is traditionally used in the treatment of rheumatism, pain, and inflammation. The present investigation evaluates the property and possible mechanism of action of the methanolic extract of* B. dalzielii *(BDME) on inflammatory and neuropathic pain models. Effects of BDME (250 and 500 mg/kg), orally administered, were verified in mechanical hypernociception induced by LPS or PGE_2_. Mechanical hyperalgesia, cold allodynia, and heat hyperalgesia were used in vincristine-induced neuropathic pain. NW-nitro-L-arginine methyl ester (inhibitor of nitric oxide synthase), glibenclamide (ATP-sensitive potassium channel blocker), methylene blue (cGMP blocker), or naloxone (opioid antagonist receptor) has been used to evaluate the therapeutic effects of BDME on PGE_2_-induced hyperalgesia. Chemical profile of BDME was determined by using HPLC-XESI-PDA/MS. BDME showed significant antinociceptive effects in inflammatory pain caused by LPS and PGE_2_. The extract also significantly inhibited neuropathic pain induced by vincristine. The antinociceptive property of BDME in PGE_2_ model was significantly blocked by L-NAME, glibenclamide, methylene blue, or naloxone. The present work reveals the antinociceptive activities of BDME both in inflammatory and in neuropathic models of pain. This plant extract may be acting firstly by binding to opioid receptors and secondly by activating the NO/cGMP/ATP-sensitive-K^+^ channel pathway.

## 1. Introduction

Because of the fact that everyone feels pain at least once in his life, pain is one of the most essential health problems in the medical field [1]. It is most often a sign of disease and acts as a warning mechanism for immediate and/or persistent tissue lesion [2]. In order to improve strategies for the management of pain, it is necessary to understand the different mechanisms that are involved in its evolution [3]. While acute pain is considered a warning sign to protect the body, chronic pain represents a disease in its own right. Several studies show that one over every three Americans suffers from chronic pain and its prevalence in Europe is 25–30% [4]. Approximately one-fifth of individuals who report chronic malaise have particular neuropathic pain [5, 6].

The limited efficacy and safety of therapeutic medications available to relieve painful conditions are the main causes of the increased prevalence of chronic and neuropathic pain [7]. Nowadays, opioids and nonsteroidal anti-inflammatory drugs (NSAIDs) are widely and increasingly used to relieve pain throughout the world [8]. The use of analgesic drugs for pain relief revealed their side effects. It is the case of opioids that cause nausea, respiratory failure, unease, and constipation. Furthermore, if applied for a long time, they can make patient develop addiction or provoke, like NSAIDs, gastrointestinal lesions or liver and renal failures [8, 9].

Due to the fact that some painful situations, particularly neuropathic pain, are rebellious to the existing analgesic drugs [10, 11] and that current treatments result in several side effects associated with a high economic cost, it is important to conduct research projects on finding new potential analgesic drugs with fewer side effects and which are cheaper that may be used in the management of such diseases [7, 12]. For decades, the development of several drugs used in modern medicine, especially to relieve pain, comes mostly from natural products [13, 14].* B. dalzielii*, belonging to the Burseraceae family is a traditional medicinal plant widely used in Cameroon. Its stem bark has been extensively solicited in traditional medicine, particularly in Africa, for the management of venereal diseases, fever, leprosy, rheumatism, ulcers, pain, inflammation, and gastrointestinal disorders [15, 16].

*B. dalzielii* is a very well-known tree, commonly called “Frankincense tree.” It has a smooth pale brown bark that is particularly separated by papery plates ragged [17, 18].* B. dalzielii* is a very tall tree (more than 13 meters high), producing small fragrant and aromatic white flowers. It belongs to the genus* Boswellia*, very widespread in many African countries, particularly in Burkina Faso, Togo, Cameroon, Benin, Ghana, Ivory Coast, and Nigeria [19, 20]. The chemical study of crude methanolic extract of the leaves of* B. dalzielii* showed the existence of flavonoids, tannins, triterpenoids, cardiac glycosides, and alkaloids while that of the bark indicates the presence of steroids, glycosides, alkaloids, triterpenoids, carbohydrates, anthraquinones, flavonoids, and saponins [15, 16, 21]. Otherwise, 4′-methoxy-(E)-resveratrol-3-O-rutinoside, incensole, *β*-sitosterol, and gallic and protocatechuic acids have been already isolated from the stem bark of* B. dalzielii* [21]. These different classes of secondary metabolites as well as some of these different compounds have been recognized to possess many pharmacological effects including analgesic effects [22, 23]. With reference to previous reports, the current study was then conducted to determine the antinociceptive properties and possible mechanism of actions of the methanolic extract of* Boswellia dalzielii* on inflammatory and neuropathic pain using animals' models.

## 2. Material and Methods

### 2.1. Plant Collection and Extraction

Fresh bark of* B. dalzielii* was harvested in Garoua (North province of Cameroon) in April 2014. After identification by comparison with a sample preserved at Cameroon National Herbarium (Yaounde, Ref: 64925/HNG/CAM), these barks were dried and crushed. Stem bark powder (280 g) was mixed with methanol (2 l) in a container tightly closed and left at ambient temperature for 72 h and then filtered. Concentration of the filtrate was made with a rotary evaporator (reduced pressure, 65°C) giving 21.17 g (9.41% yields).

### 2.2. LC-MS Analysis of BDME

For the analysis, 0.2 g of BDME was weighted and dissolved in extraction solvent (methanol-water (70 : 30, v/v)). After that, it was filtered through 0.45 *μ*m membrane filter prior to the injection into HPLC-MS system. HPLC-MS system was a LC-binary pump with PDA detector (*λ*_max_ = 500 nm), autosampler (injection volume 5 *μ*l), and thermostated column (Merck C-18.55 mm × 4 mm × 34 mm) (40°C). The mobile phase was prepared using 0.1% formic acid in water for A and 0.1% formic acid in methanol for B. The flow rate was 0.5 mL/min. MS QQQ Mass Spectrometer (operating in positive mode) equipped with a XESI ion source was used for measuring mass spectra. Data were integrated by Trilution LC software and the results were obtained by comparison with standard.

### 2.3. Phytochemical Screening of BDME

The method described by Matos [24] has been used to verify the presence of chemical compounds belonging to the class of tannins, anthraquinones, alkaloids, saponins, steroids, triterpenoids, flavonoids, and so forth. The tests were based on the visual observations of a change in color or formation of precipitate after the addition of specific reagents. 

*Tannins*. About 2 ml of the extract was stirred with 2 ml of distilled water and few drops of FeCl_3_ solution were added. The formation of green precipitate was an indication of the presence of tannins. 

*Saponins*. 5 ml of extract was shaken vigorously with 5 ml of distilled water in the test tube and warmed. The formation of stable foam was an evidence for the presence of saponins. 

*Flavonoids*. To 1 ml of extract 1 ml of 10% lead acetate solution was added. The formation of a yellow precipitate was taken as positive test of flavonoids. 

*Anthraquinones*

- 3 ml of extract was shaken with 3 ml of benzene and filtered and 5 ml 10% ammonia solution was added to the filtrate. The mixture was shaken and the presence of a pink, red, or violet color in the ammoniacal (lower) phase indicates the presence of free anthraquinones.
- 3 ml of extract was boiled with 3 ml of sulphuric acid and filtered while hot. 3 ml of benzene was added to filtrate and shaken. The benzene layer was separated and 3 ml of 10% ammonia solution was added. A pink, red, or violet color in the ammoniacal (lower) phase indicates the presence of anthraquinones derivatives.

*Terpenoids*. 2 ml of the extract was dissolved in 2 ml of chloroform and evaporated to dryness. 2 ml of concentrated sulphuric acid was then added and heated for about 2 min. A grayish color indicated the presence of terpenoids. 

*Steroids*. The development of a greenish color when 2 ml of the extract was dissolved in 2 ml of chloroform and treated with sulphuric and acetic acids indicated the presence of steroids. 

*Alkaloids*. The presence of alkaloids was put in evidence by adding Mayer's and Wagner's reagents on the mixture of 3 ml of extract stirred with 3 ml of 1% HCl. The turbidity of the resulting precipitate was taken as positive test of alkaloids and so forth.

### 2.4. Experimental Animals

In the present study, male and female* Wistar* rats were used for anti-inflammatory assays while only female* Wistar* rats were used for neuropathic pain tests. All animals were weighing between 150 and 200 g with 10 to 12 weeks of age. Animal House of H.E.J. Research Institute of Chemistry, International Center for Chemical and Biological Sciences (ICCBS), University of Karachi, Pakistan, provided animals. During 1-week acclimatization (22 ± 1°C temperature and 50–80% humidity), with 12 hours' light/dark cycle and freely receiving standard diet for rodents and filtered water beforehand, effects coherence of administered treatments was determined using a minimum possible number of rats. Animals treatment was in agreement with the Institutional Animal Care, Use and Standards Committee (IACUC) of ICCBS (Protocol Number 1209004), and study protocol was accepted by the ethics committee of ICCBS, University of Karachi, Pakistan.

### 2.5. Behavioral Testing

#### 2.5.1. Hindpaw Withdrawal Response Induced by Analgesiometer

Analgesiometer (UGO Basile, Italy) is a device for determining animal's response on mechanical pain threshold after pressure exerted on the paw. For this study, animals were acclimatized for 1 h before behavioral testing and mechanical hypersensitivity was evaluated at several periods depending on the test used. The machine was programmed to exert an increasing force of 0–400 g, with 400 g considered as maximum pressure force applied to avoid paws damage [25].

#### 2.5.2. Treatment Regimen and LPS Induced Hyperalgesia

The basal reaction was measured with analgesymeter as previously described and animals received orally vehicle (10 ml/kg), diclofenac (50 mg/kg) or BDME (250 and 500 mg/kg). One hour after treatment, animals were injected with LPS (100 ng/paw,* i.pl.*). The nociceptive threshold corresponded to the time when the rats vocalize or struggle energetically and was estimated by the average of three consecutive pressure tests recorded before injection of LPS (zero time, 100%) and 1, 2, 3, 4, 5, and 6 h after LPS injection, which represented the peak of effect. Extent of hyperalgesia was estimated as a variation between pretreatment and peak of effect pressure test averages (Δ of nociceptive threshold) and expressed as % [26, 27].

#### 2.5.3. PGE_2_-Induced Mechanical Hyperalgesia

Experimentation was realized as previously described by Kassuya et al. [28]. Animals received orally vehicle, BDME, or diclofenac at respective dose of 10, 500, or 50 mg/kg PGE_2_ (0.1 nmol/paw,* i.pl.*) which was injected 1 hour later. Frequency response to paw pressure stimulation was measured before PGE_2_ injection and 1, 2, 3, 4, 5, and 6 hours after that [29]. In order to examine whether the different treatments act on PGE_2_ receptors or on their downstream, animals were given extracts and substances 1 h after injection of PGE_2_* (i.pl.)* [30] and hypernociception was estimated at the same periods.

#### 2.5.4. Involvement of NO/cGMP/K^+^ Pathway or Participation of Endogenous Opioids

Concerning action mechanism of BDME, posttreatment effects were evaluated in the absence and in the presence of L-NAME (90 mg/kg,* i.p.*), an inhibitor of NO production, methylene blue (1 mg/kg,* i.p.*), a soluble guanylyl cyclase inhibitor, or glibenclamide (5 mg/kg,* i.p.*), an inhibitor of ATP-sensitive potassium channels [30, 31]. These antagonists made the verification of NO/cGMP/potassium channel contribution possible, in the antinociceptive property of BDME. Antagonists or their respective vehicles were administered 20 min before that of BDME (500 mg/kg,* p.o.*) or vehicle (10 mg/kg,* p.o.*). Each animal was used once for experimentation and all treatments were given 60 min before PGE_2_ injection. Evaluation of the threshold after mechanical nociceptive pressure exerted using the analgesiometer allowed the determination in the extent of hyperalgesia at 3 and 4 hours after PGE_2_ injection.

#### 2.5.5. Induction of Neuropathic Pain with Vincristine

To induced neuropathy in Sprague-Dawley rats, vincristine sulphate (100 *μ*g/kg) was administrated* (i.p.)* into the hindpaw in two series of 5 successive working days (1, 2, 3, 4, and 5 days and then 8, 9, 10, 11, and 12 days) [32]. 30 min after vincristine injection, reaction sensitivity to pain materialized by vocalizations and/or paw withdrawals was measured using the Randall-Selitto test.

#### 2.5.6. Treatment and Behavioral Assessment of Neuropathic Pain

Evaluation of BDME effect in vincristine-induced neuropathic pain, analgesiometer test (mechanical hyperalgesia), hotplate test (thermal hyperalgesia), and cold allodynia (cold water (4°C)) were conducted on days 0, 5, 10, 14, 15, 16, 17, 18, 19, and 20. On the 15th day, after having distributed rats presenting signs of neuropathy in 3 groups of 6 each, being treated with BDME (500 mg/kg), morphine (5 mg/kg), or vehicle (5% DMSO), respectively, each rat was subjected to the sensitivity pain 0.5, 1, 2, 4, 6, and 8 hours after treatment. They were further given a daily dose of treatment until the 20th day and hyperalgesia was also evaluated daily.

Mechanical hyperalgesia using the analgesiometer was performed as described earlier [25]. Thermal hyperalgesic test was executed in rats after the vincristine-induced neuropathy using the hot plate analgesiometer (51 ± 0.5°C) with cut-off latency of 20 s [33, 34].

Cold allodynia was assessed by immersing (cold water, 4°C) rat's tails and tail withdrawal latency was measured with a digital timer. Immersion duration was recorded with a cut-off time of 15 s [35, 36].

#### 2.5.7. Biochemical Estimations

On day 21, animals were anaesthetized with anesthetic ether and blood was withdrawn through cardiac puncture and introduced into a tube containing EDTA as anticoagulant and into another tube without anticoagulant. Hematological parameters like WBC (white blood cell) count, Hb (haemoglobin), PLT (Hematocrit and platelets), and RBC (red blood cell) count were determined in blood with anticoagulant by usual standard laboratory methods [37]. Otherwise, blood without anticoagulant was centrifuged (4900 rpm for 5 minutes), the serum was collected, and serum AST, ALT, ALP, and creatinine levels were also measured [38].

#### 2.5.8. Chemicals and Drugs

L-NAME, MB (methylene blue), glibenclamide, prostaglandin E_2_, diclofenac, morphine, naloxone chlorhydrate, and DMSO (dimethylsulfoxide) were acquired from Sigma (Sigma Chemical Co., St. Louis, MO, USA). MB, diclofenac, morphine, and glibenclamide were dissolved in 5% DMSO (DMSO + PBS) whereas L-NAME, diazoxide, prostaglandin E_2_, and naloxone chlorhydrate were dissolved in saline.

### 2.6. Statistical Analysis

Results were expressed as mean ± standard error of mean (*n* = 6). Data results from behavioral and biochemical tests were statistically analyzed by using GraphPad Prism version 5.0.1 software for Windows. Groups of data were analyzed by either one-way ANOVA followed by Tukey post hoc test or two-way ANOVA followed by Bonferroni post hoc test. *p* value * *<*  * 0.05 was considered statistically significant in all analyses.

## 3. Results

### 3.1. HPLC Analysis and Phytochemistry Screening

**Figure 1:**
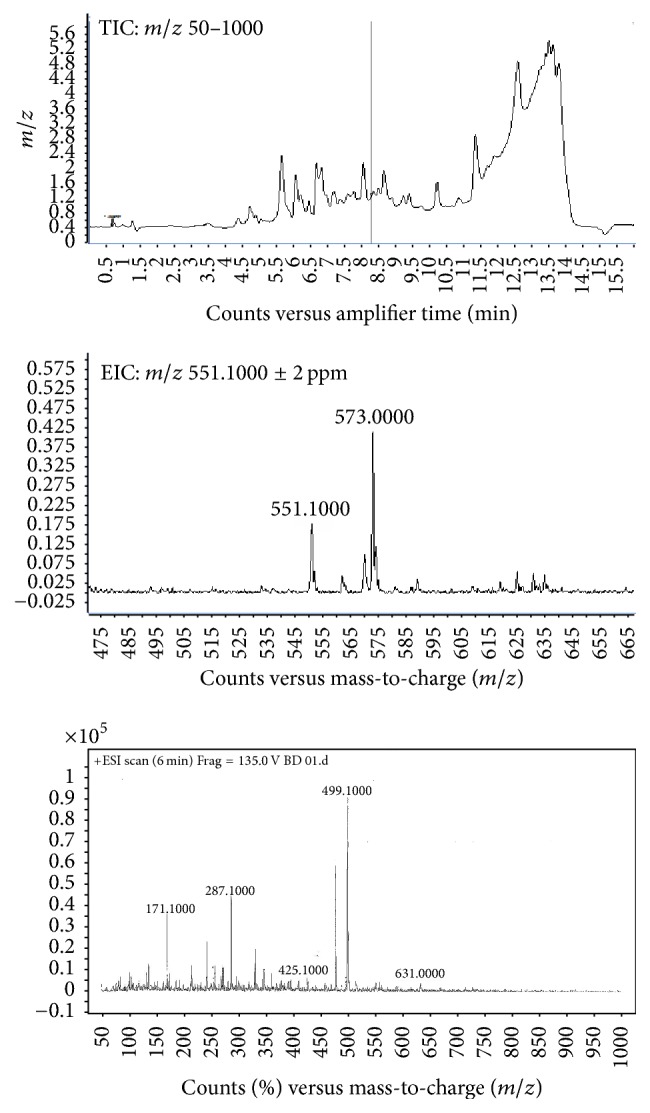
Total ion current (TIC) and extracted ion chromatograms of incensole and gallic acid.

Results of this screening showed that alkaloids, flavonoids, cardiac glycosides, steroids, triterpenoids, tannins, and saponins are present in the BDME (Table 1). Analysis of this extract using LC-MS exhibits several compounds. Besides the various signals in the total ion chromatogram (TIC) (*m/z* 50–1000), the masses obtained from the ion chromatograms (±2 ppm) of particular quasi molecular ions (*m/z* 171.1000;* m/z* 551.1000) represented the possible phenolics compounds which may correspond to gallic acid and incensole, respectively (Figure 1).

### 3.2. Effects of BDME on Mechanical Hypernociception Induced by LPS

**Figure 2:**
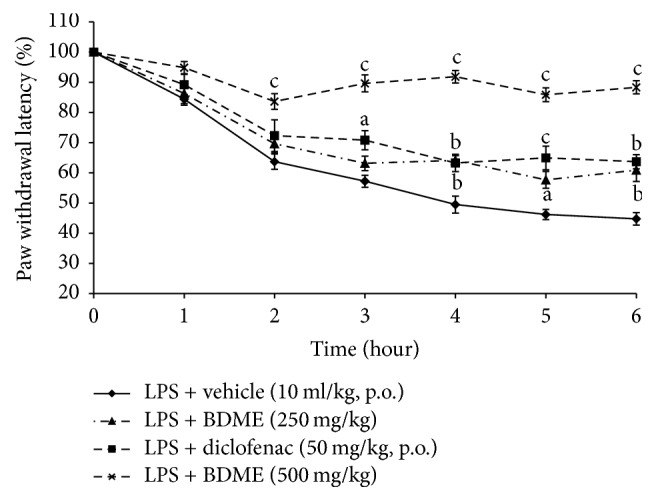
Effect of oral administration of the methanol extract of the stem bark of* Boswellia dalzielii* (BDME) on the mechanical hypernociception assessed with analgesymeter in mechanical hypernociception induced by* i.pl. *injection of LPS (100 ng/paw). *n* = 6. ^a^*p* < 0.05; ^b^*p* < 0.01; ^c^*p* < 0.001, significantly different compared to vehicle.

Injection of LPS produced important and gradual increases in rat hypersensitivity to paw pressure stimulation. BDME orally administered reduced significantly (*p* < 0.001) mechanical hypernociception induced by LPS. This effect is significant 2 hours after LPS injection and is maintained until the 6th hour. Maximum effect of BDME was observed at the 3rd and 4th hour with 75.47% and 83.52% of inhibition, respectively (Figure 2).

### 3.3. Effects of BDME on PGE_2_-Induced Mechanical Hypernociception

**Figure 3:**
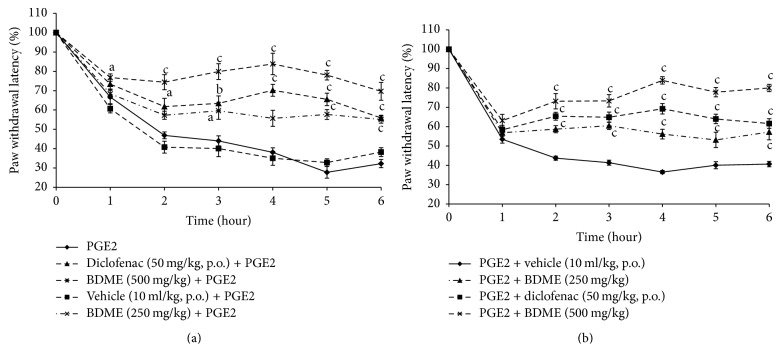
Effect of oral administration of the methanol extract of the stem bark of* Boswellia dalzielii* (BDME) on the mechanical hyperalgesia in PGE_2_ (100 *μ*l) inflamed rat paw. In panel (a), animals were treated 1 hour before PGE_2_ injection and pain response was evaluated before treatment and after PGE_2_ injection. In panel (b), treatments were given orally 1 hour after PGE_2_ and response to pain was evaluated before PGE_2_ injection and after treatment. *N* = 6; ^a^*p* < 0.05; ^b^*p* < 0.01; ^c^*p* < 0.001 significantly different compared to vehicle.

Injection of PGE_2_ (1 h before treatment) induced a remarkable mechanical hypersensitivity. This hypernociception was considerably reduced by diclofenac (50 mg/kg,* p.o.*) and BDME (500 mg/kg,* p.o.*) in preventive treatment with inhibitions of 54.95 and 75.04% (*p* < 0.001), respectively, at 4th hour (Figure 3(a)). In the second case, curative treatment with both BDME and diclofenac significantly inhibited hypernociception caused by PGE_2_ and their effects was significant (*p* < 0.001) 2 hours after its administration and maintained until the 6th hour, with the maximum effect observed at the 4th hour (51.33 and 73.84%, resp., for diclofenac and BDME) (Figure 3(b)).

### 3.4. Effects of L-NAME and L-Arginine on the Antinociceptive Effect of BDME

**Figure 4:**
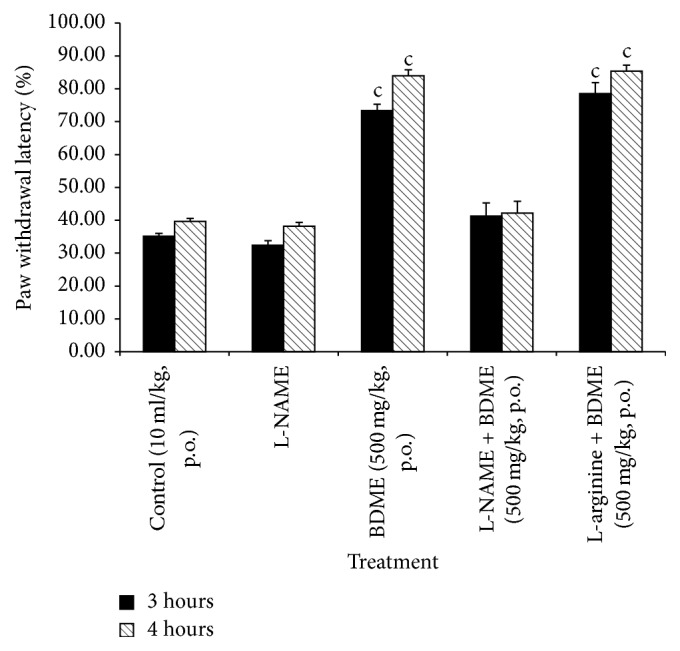
Effect of L-NAME (90 mg/kg,* i.p.*) and L-arginine (200 mg/kg,* i.p.*) on oral administration of the methanol extract of the stem bark of* Boswellia dalzielii* (BDME) on the mechanical hyperalgesia in PGE_2_ inflamed rat paw. *N* = 6. ^c^*p* < 0.001 significantly different compared to control.

As shown in Figure 4, intraperitoneal* (i.p.)* injection of L-NAME (90 mg/kg) had no important influence by themselves on the mechanical hypernociception induced by injection of PGE_2_ (0.1 nmol/paw). But, given 15 min before treatment, L-NAME considerably reduced the antinociceptive activity of the BDME (500 mg/kg,* p.o.*), while L-arginine (200 mg/kg,* i.p.*), injected 15 min before treatment, enhanced antinociceptive effect of BDME.

### 3.5. Effects of Glibenclamide and Methylene Blue (MB) on the Antinociceptive Effect BDME

**Figure 5:**
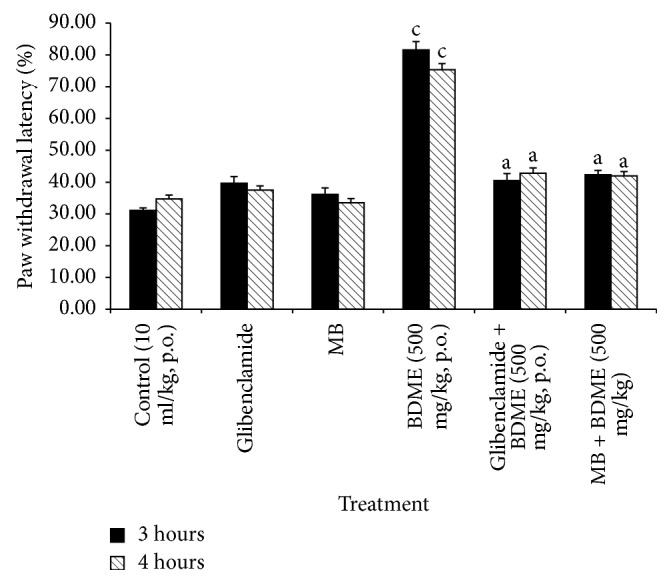
Effect of glibenclamide (2 mg/kg,* i.p.*) and methylene blue (MB) (20 mg/kg,* i.p.*) on oral administration of the methanol extract of the stem bark of* Boswellia dalzielii* (BDME) on the mechanical hyperalgesia in PGE_2_ inflamed rat paw. *N* = 6. ^a^*p* < 0.05; ^c^*p* < 0.001, significantly different compared to control.

As shown in Figure 5, intraperitoneal* (i.p.)* injection of glibenclamide (5 mg/kg) or MB (5 mg/kg) had no important influence by themselves on the mechanical hypernociception induced by PGE_2_ (0.1 nmol/paw,* i.pl.* injection). However, given 15 min before treatment, both substances considerably reduced the antinociceptive activity of the extract of BDME (500 mg/kg,* p.o.*).

### 3.6. Effects of Naloxone on the Antinociceptive Effect BDME

**Figure 6:**
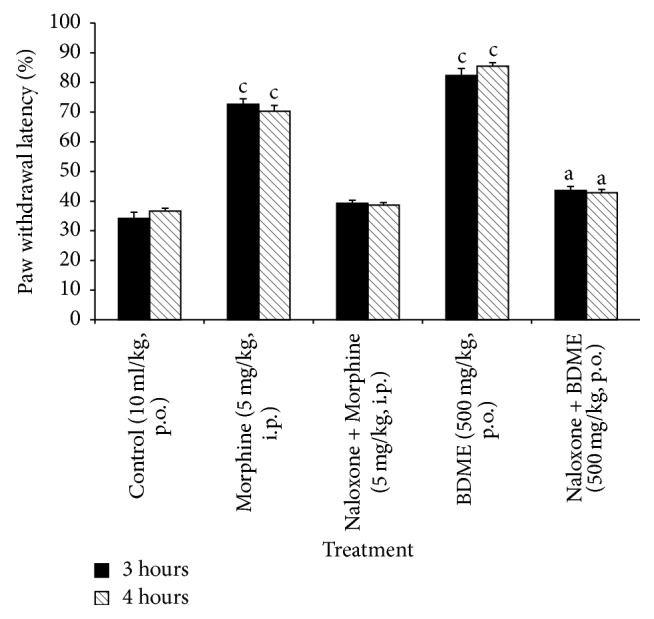
Effect of naloxone (20 mg/kg,* i.p.*) on oral administration of the methanol extract of the stem bark of* Boswellia dalzielii* (BDME) on the mechanical hyperalgesia in PGE_2_ inflamed rat paw. *N* = 6. ^a^*p* < 0.05; ^c^*p* < 0.001 significantly different compared to control.

Figure 6 showed that naloxone, an endogenous opioids receptors antagonist, given 15 min before treatment, significantly reduced the antinociceptive effects of the BDME (500 mg/kg,* p.o.*) and of morphine (5 mg/kg,* i.p.*).

### 3.7. Effect of BDME in Neuropathic Pain Induced by Vincristine

#### 3.7.1. Induction of Neuropathic Pain by Vincristine

**Figure 7:**
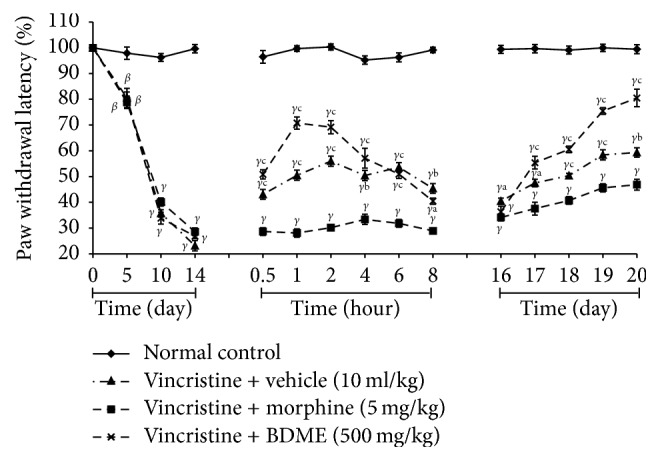
Effect of methanol extract of the stem bark of* Boswellia dalzielii* (BDME) on mechanical hyperalgesia in vincristine-induced neuropathic pain. Data were expressed as mean ± SEM, *N* = 6 rats per group. ^*β*^*p* < 0.01; ^*γ*^*p* < 0.001 significantly different compared to normal control; ^a^*p* < 0.05; ^b^*p* < 0.01; ^c^*p* < 0.001 significantly different compared to vincristine and vehicle group.

**Figure 8:**
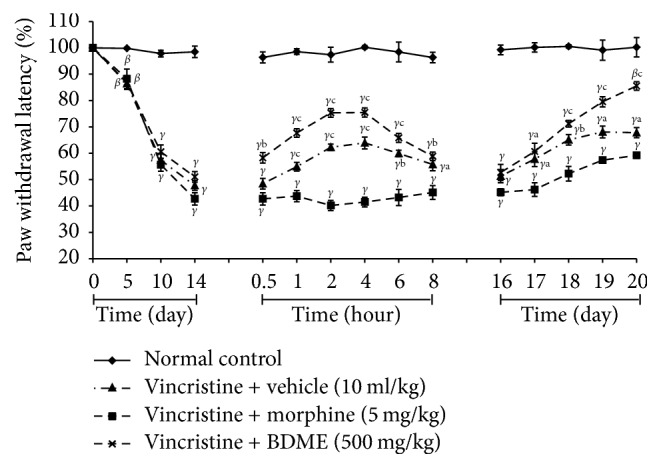
Effect of methanol extract of the stem bark of* Boswellia dalzielii* (BDME) on paw heat hyperalgesia in vincristine-induced neuropathic pain. Data were expressed as mean ± SEM, *N* = 6 rats per group. ^*β*^*p* < 0.01; ^*γ*^*p* < 0.001 significantly different compared to normal control; ^a^*p* < 0.05; ^b^*p* < 0.01; ^c^*p* < 0.001 significantly different compared to vincristine and vehicle group.

**Figure 9:**
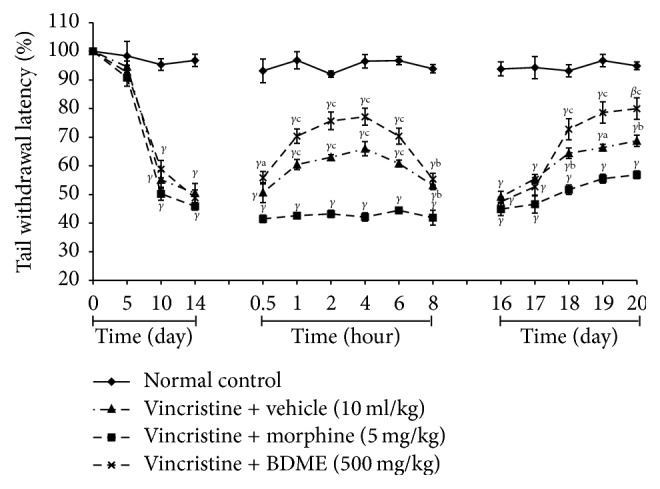
Effect of methanol extract of the stem bark of* Boswellia dalzielii* (BDME) on tail cold hyperalgesia in vincristine-induced neuropathic pain. Data were expressed as mean ± SEM, *N* = 6 rats per group. ^*β*^*p* < 0.01; ^*γ*^*p* < 0.001 significantly different compared to normal control; ^a^*p* < 0.05; ^b^*p* < 0.01; ^c^*p* < 0.001 significantly different compared to vincristine and vehicle group.

An assessment of the paw withdrawal latencies to mechanical, heat, and tail cold hyperalgesia during 10-day induction of neuropathic pain yielded a gradual decline of the latencies from day 1 to day 12. There was no important modification between the various designated groups during the induction period. However, there was significant decline of the paw withdrawal latencies from day 5 to day 14 on all experimental groups compared to normal control group, suggesting a reduced pain threshold and an induction of peripheral neuropathy by day 14 (Figures 7, 8, and 9).

#### 3.7.2. Mechanical Hyperalgesia

BDME (500 mg/kg) produced significant (*p*  < 0.05) analgesic effect in the Randall-Selitto mechanical hyperalgesic test on day 15. This activity is significant (*p* < 0.001) 0.5 hours after treatment and was maintained significant (*p* < 0.001) for the next 8 hours, but BDME did not reveal any activity when given once per day and estimated the next day. However the important result produced by BDME was preserved throughout the experiment when it was evaluated 1 hour after the first treatment of each day (Figure 7).

#### 3.7.3. Cold Allodynia

BDME (500 mg/kg) increased the latency to tail withdrawal in the cold water and produced significant (*p* < 0.001) antiallodynic effect on day 15 (0.5, 1, 2, 4, 6, and 8 hours after treatment) and 1 hour after the first administration on days 17, 18, 19, and 20. The overall anticold allodynic effect of BDME was not significant on day 16 (Figure 8).

#### 3.7.4. Thermal Hyperalgesia

On day 15, there was an important modification between the BDME-treated rats and the vehicle control group. Results revealed important effects at 0.5, 1, 2, 4, 6, and 8 hours after BDME treatment and 1 hour after the first administration on days 18, 19, and 20. The overall antihyperalgesic effect of BDME was not significant on days 16 and 17 (Figure 9).

#### 3.7.5. Effect of BDME Extracts on Hematological and Serum Parameters

The significant (*p* < 0.001) increase levels of platelets and WBC, and the significant (*p* < 0.001) decrease levels of RBC, Hb, and hematocrit were observed in control group. However, the treatment with BDME (500 mg/kg) and morphine (5 mg/kg) revealed an important increase (*p* < 0.001) in the Hb, RBC, and hematocrit. Additionally, BDME (500 mg/kg) considerably (*p* < 0.001) attenuated the increase of platelets and WBC. Finally, the average value was comparatively near that of normal animals (Table 2).

#### 3.7.6. Effect of BDME Extracts on Biochemical Parameters

As a result of vincristine-induced neuropathic pain, the serum levels of AST, ALT, ALP, and creatinine were increased significantly in control group. These enzyme levels were altered by treatment with PAME and morphine. The levels of AST, ALT, ALP, and creatinine were significantly (*p* < 0.001) decreased by treatment with BDME (500 mg/kg) and morphine (5 mg/kg) (Table 3).

## 4. Discussion

This study was proposed with the aim of determining antinociceptive properties and a possible action mechanism of the methanolic extract of the bark of* Boswellia dalzielii* using inflammatory and neuropathic pain in rats model. It has been demonstrated that methanolic extract, given orally, induces antinociceptive properties when tested against the development of hypernociception induced by LPS and PGE_2_ (mechanical inflammatory pain), as well as neuropathic pain induced by vincristine. Results also show that L-NAME, glibenclamide, methylene blue, and naloxone significantly inhibited the antinociceptive effects elicited by* Boswellia dalzielii* extract when assessed in PGE_2_ induced mechanical hypernociception.

Oral administration of BDME significantly inhibited inflammatory pain induced by LPS, which is a constituent of the external membrane of Gram-negative bacteria identified as an effective stimulator of the immune system. It is able to induce several genes expression in different inflammatory cell types [39]. Indeed, the intraplantar injection of LPS causes mechanical hypersensitivity via the stimulation of proinflammatory mediators release like tumor necrosis factor-alpha (TNF-*α*), interleukin-6 (IL-6), PGE_2_, and interleukin-1 (IL-1) [40], which contribute to peripheral nervous system modulation [41]. In addition, LPS activates some receptors that cause IK-B phosphorylation and nuclear factor-kappa B (NF-kB) translocation, which leads to the production of multiple proteins comprising TNF-*α*, IL-1*β*, IL-6, bradykinin B1 receptors, inducible nitric oxide synthase (iNOS), and/or cyclooxygenase-2 (COX-2) [42]. It is known that peripheral tissue inflammation causes persistent pain behavior in animals, identically as in clinical states of inflammatory pain [43]. Thus, antihypernociceptive effect of BDME seems to be associated with a local anti-inflammatory action.

These considerations led to analyze whether the antinociceptive effects of BDME were associated with its ability to prevent the synthesis and/or the activity of inflammatory mediators. Injection of PGE_2_ can sensitize nociceptors to chemical and mechanical stimuli [44] or increase the excitability of nociceptive sensory neurons with an increase of sodium current at the level of tetrodotoxin-resistant channels (TTXr) [45, 46] and a suppression of potassium currents [45, 47, 48]. BDME, given before or after PGE_2_ injection, exhibited important antinociceptive activity, attesting that its action is not only at the level of COX and implying the participation of the PGE_2_ pathway. These results lead to believe that BDME do not act on prostaglandin receptors but could act on the cascade of biochemical reactions triggered by the activation of PGE_2_-receptors.

It can then be thought that BDME produces its effect by acting on the process of mechanical pain installation in inflammatory conditions and could also effectively affect well-established chronic pain. This second theory was assessed by evaluating the effect of BDME on neuropathic pain induced by vincristine. In neuropathic pain model induced by vincristine, NF-kB is activated and the level of TNF-*α* increases in the sciatic nerve [49, 50]. A high level of TNF-*α* is observed in the spinal cord of mice in the vincristine-induced allodynia model, but treatment with neutralizing antibody of TNF-*α* reduced significantly vincristine-induced mechanical allodynia [51]. Also, tactile allodynia, the vincristine-induced neuropathic pain on rat model, is significantly reduced when given a drug agent that inhibits or blocks IL-1*β* production [52]. Like in LPS induced inflammatory hypernociception, BDME considerably reduced the well installed neuropathic pain induced by vincristine. On this type of pain generally known as rebel to medications, BDME significantly reduced hyperalgesia (mechanical, cold, and heat) 0.5 hours after its oral administration and this activity remained significant until the sixth hour. However, in this model of neuropathic pain, the extract did not show a long-term activity. In fact, after one administration per day, the antinociceptive effect could not be observed within 24 h. Nevertheless, the effect of BDME appears after being administered once a day for several days. It follows from these results that BDME exerts antinociception effects by inhibiting the endogenous production of proinflammatory cytokines (TNF-*α* and/or IL-1*β*) and/or other mediators of inflammation and these effects are mediated through the regulation of NF-kB [53].

For patients with normal hepatic function and under anticancer therapy, severe hepatitis may develop [54]. Otherwise, Harrison [55] has shown that administration of vincristine in mice causes weight loss, elevated plasma alkaline phosphatase, ALT, and AST activities, and granulocytopenia and reticulocytopenia. It is clear from our investigation that platelets, WBC, AST, ALT, ALP, and creatinine levels have increased significantly, while RBC, Hb, and hematocrit have significantly decreased in nontreated group. BDME and morphine treated group improves significantly the level of all hematological (platelets, WBC, RBC, Hb, and hematocrit) and biochemical (AST, ALT, ALP, and creatinine) parameters compared to nontreated group. These results indicate that, in addition to protecting against the development of neuropathic pain, BDME can also prevent side effects development (hematologic and hepatic) related to the administration of vincristine. Indeed, vincristine administration in patients results in bone marrow suppression and severe hepatic injury [56, 57]. The biochemical restoration observed in this study may be due to the effect of BDME on the regulation of immune system and/or its hepatoprotective properties, which may be related to the antioxidant properties of BDME or to its inhibitory effect on cytochrome P_450_ or to the inhibition of the synthesis of enzyme responsible for injury and inflammation of hepatocytes [58]. Thus this plant can be a valuable asset to prevent the appearance of side effects in patients suffering from cancer and under chemotherapy.

In another register, after injection of PGE_2_, it directly sensitizes the neurons responsible for peripheral nociception [59, 60] and stimulates cytokine release and the production of other mediators [61, 62]. It is known that, in hypernociception (inflammatory pain), nitric oxide (NO) is able to activate the guanylate cyclase enzyme, which is directly responsible for an increase in intracellular levels of cyclic guanosine monophosphate (cGMP) [63]. Then, cGMP induces the opposite effect of cyclic adenosine monophosphate (cAMP) [64] and promotes antihypernociception. Sachs et al. [65] proved that stimulation of protein kinase G by cGMP is essential for the opening of ATP-sensitive-K^+^ channels (K_ATP_) in analgesia. Membrane hyperpolarization occurs as a result of the prolonged opening of the ATP-sensitive-K^+^ channels, and this leads to a decrease in depolarization capacity at the level of the neuron membrane, followed by a reduction in the action potential transmission and consequently production of an analgesic effect [1]. This mechanism is one of the many molecular events which condition the appearance of painful phenomena [66, 67]. In addition, opioid agonists such as morphine [68] cause the opening of ATP-sensitive potassium channels after their binding to opioid receptors.

In the remainder of this study, we research the action mechanisms by which BDME induce their analgesic activities. For it, we use several types of antagonists. L-NAME (inhibitor of nitric oxide synthase), glibenclamide (antagonist of ATP-sensitive-K^+^ channels), methylene blue (cGMP blocker), and naloxone (endogenous opioids receptor antagonist) have been used in pain model induced by PGE_2_. These results indicate that antinociceptive effect of BDME is probably linked to opioid system, by activation of opioid receptor (direct action) or by stimulation of endogenous opioid release (indirect action), which leads to NO production, guanylate cyclase activation, and ATP-sensitive-K^+^ channel activation [69]. Phytochemical study revealed existence of saponins, flavonoids, tannins, and triterpenes. Flavonoids and triterpenes have already proved in many previous studies an important antinociceptive activity [70–73] and several of these compounds produce this activity by binding to opioid receptors followed by activation of NO/cGMP pathway with K_ATP_-channel opening [74, 75]. The capacity of flavonoids and triterpenes to react on this pathway could justify the antinociceptive activity of the extract. These observations reinforce our position that BDME produces its antinociceptive effect by acting on opioid receptors and activation of the NO/cGMP/K^+^ channel pathway. In addition, recent reports have shown that, among the compounds already isolated in* B. dalzielii*, phenolic compounds such as gallic acid [21] are present, and the HPLC profile realized in this study confirms well the presence of this compound in BDME. This chemical compound revealed potent antinociceptive activity against inflammatory and neuropathic pain model induced, respectively, by carrageenan (*i.pl.* injection) and chronic constriction injury of nerve [23]. For this reason, the antinociceptive property revealed by BDME in this work can be justified in part by the presence of this compound and a possible synergistic action between the other components present in the extract. In order to ensure the appropriate medical use of* Boswellia dalzielii*, further studies are needed to establish the possible relationship between its activity and its chemical composition.

## 5. Conclusion

Data from this study indicated that the methanol extract from the stem bark of* Boswellia dalzielii* possesses antinociceptive effects against inflammatory nociception as well as against neuropathic pain. This plant extract may be acting firstly by binding to opioid receptors and secondly by activating the NO/cGMP/ATP-sensitive-K^+^ channel pathway. This activity is due to an isolated action of many compounds present in the plant or has a synergistic effect of these different compounds. Therefore, it seems logical to propose that this vegetal extract be recognized as a new therapeutic opportunity for the management of painful states.

## Figures and Tables

**Table 1:** Phytochemical profile of BDME.

Metabolites	1	2	3	4	5	6	7	8	9	10
BDME	++	++	++	+	−	+	+	++	+	+

−: absent; +: trace; ++: present in appreciable quantity; 1: alkaloids, 2: flavonoids, 3: steroids, 4: triterpenoids, 5: anthraquinones, 6: tannins, 7: saponins, 8: cardiac glycosides, 9: carbohydrates, and 10: phenol.

**Table 2:** Influence of BDME on blood parameters after vincristine-induced neuropathic pain in rats.

	Dose (mg/kg)	Haemoglobin (g/dl)	RBC (million/*µ*l)	Hematocrit (%)	WBC (10^9^/L)	Platelet count (10^9^/L)
Normal control	-	12.45 ± 0.25	7.06 ± 0.19	40.38 ± 1.11	7.58 ± 0.28	831.75 ± 34.48
Vincristine + vehicle	-	7.15 ± 0.39^*γ*^	3.90 ± 0.19^*γ*^	25.93 ± 1.03^*γ*^	1.68 ± 0.14^*β*^	481.25 ± 26.80^*β*^
Vincristine + Morphine	5	11.60 ± 0.35^c^	6.05 ± 0.52^*α*c^	35.10 ± 1.27^*α*b^	9.00 ± 0.52^c^	1074.00 ± 15.01^*α*c^
Vincristine + BDME	500	12.65 ± 0.55^c^	6.87 ± 0.14^c^	39.68 ± 1.16^c^	8.13 ± 0.49^c^	1001.00 ± 58.87^c^

RBC: red blood cell; WBC: white blood cell; Hb: haemoglobin. Each value represents the mean ± ESM for six animals and analyzed by two-way ANOVA followed by Tukey post hoc test. ^*α*^*p* < 0.05; ^*β*^*p* < 0.01; ^*γ*^*p* < 0.001 statistically significant compared to normal control. ^c^*p* < 0.001 statistically significant compared to vincristine and vehicle. ^b^*p* < 0.01.

**Table 3:** Effect of BDME on serum parameters in vincristine induced neuropathic pain in rats.

	Dose (mg/kg)	ALP (U/l)	ALT (U/l)	AST (U/l)	Creatinine (mg/dl)
Normal control	-	72.80 ± 1.83	41.20 ± 2.56	44.00 ± 2.03	0.41 ± 0.03
Vincristine + vehicle	-	481.00 ± 9.68^*γ*^	182.00 ± 7.23^*γ*^	136.00 ± 3.03^*γ*^	0.87 ± 0.01^*γ*^
Vincristine + Morphine	5	186.00 ± 27.70^*γ*c^	85.00 ± 4.18^*γ*c^	97.00 ± 3.03^*γ*c^	0.58 ± 0.03^*γ*c^
Vincristine + BDME	500	87.00 ± 2.68^c^	48.40 ± 2.16^c^	74.40 ± 2.23^*γ*c^	0.41 ± 0.03^c^

ALP: alkaline phosphatase; AST: aminotransferase; ALT: alanine aminotransferase. Each value represents the mean ± ESM for six animals and analyzed by two-way ANOVA followed by Tukey post hoc test, ^*γ*^*p* < 0.001 when compared to normal control, ^c^*p* < 0.001 when compared to vincristine and vehicle.
